# If you cannot see it, is it still there?

**DOI:** 10.1107/S160057672500130X

**Published:** 2025-02-28

**Authors:** Lukas Leitner, Jesse Hudspeth, Sebastiaan Werten, Bernhard Rupp

**Affiliations:** a Institute of Genetic Epidemiology Medical University of Innsbruck Schöpfstrasse 41 6020Innsbruck Austria; b Department of Biochemistry and Molecular Genetics University of Colorado Anschutz Medical Campus 13001 East 17th Place Aurora CO80045 USA; c Department of General, Inorganic and Theoretical Chemistry University of Innsbruck Innrain 80-82 6020Innsbruck Austria; Instituto Andaluz de Ciencias de la Tierra, Granada, Spain

**Keywords:** ensemble refinement, modelling, invisible protein segments, flexibility, missing electron density, protein crystallography

## Abstract

Protein crystallographers rely on electron density to build atomic models of molecular structures, yet flexible regions often remain unseen in electron density and are omitted. We suggest that ensemble refinement can be used to visualize and analyse the conformational landscape of such ‘invisible’ protein segments, which is particularly useful in cases where molecular flexibility plays a functional role. Using ensemble refinement on multiple crystal forms of the fungal methyl­transferase PsiM as an example, we illustrate the dynamic nature of a key substrate recognition loop, demonstrating its potential role in substrate binding and release. Ensemble refinement provides a persuasive visualization of biologically relevant flexible regions and can be a powerful tool for exploring molecular plasticity and aiding the modelling of dynamic protein components.

## Abstract 2

Flexible segments of protein molecules, while known to be present in a structure, are often not visible in the electron density and are thus omitted in models. Ensemble refinement of completed models allows for visualization and exploration of the available conformation space of such ‘invisible’ regions. Combined with the analysis of different crystal forms, this enables insights into functional aspects of molecular plasticity to be inferred.

## 1. The problem

### 1.1. Preamble

X-ray crystallography is an experimental technique for molecular structure determination (Blundell & Johnson, 1976 ▸; Rhodes, 2006 ▸; Rupp, 2009 ▸). The primary crystallographic evidence is electron density, into which an atomic model is built. These seemingly innocuous facts imply an important distinction: we are not determining an absolute (or ‘real’) molecular structure; instead, we are providing a static model that approximates the actual structural ensemble within a specific crystalline environment. This environment imposes various restrictions on the conformational freedom of the molecule, that is, the environment determines or limits the available conformational space of the molecules.

### 1.2. Seeing is believing

Reliance on electron density as the primary crystallographic evidence for model building carries clear benefits: electron density provides ‘proof positive’ that a given molecular arrangement of atoms is present at a given location and in a specific conformation (*i.e.* a defined molecular pose). This epistemological concept of proof positive is extraordinarily important (*e.g.* Pozharski *et al.*, 2013 ▸) for general situations in which *non-covalently* linked entities such as substrates, coenzymes, inhibitors or other ligands are of interest. A model built with high confidence into clear electron density can provide the basis for structure–function relations in chemical or mechanistic models such as enzyme action or functional inhibition by therapeutic (or recreational, Section 5) drugs. On the other hand, indeterminate or spurious electron density opens the possibility for wishful overinterpretation and fanciful models (Bacon, 1620 ▸; Kleywegt & Jones, 1995 ▸), often with unfavourable consequences for proposers of unsubstantiated claims based on unverifiable structure models (Wlodawer *et al.*, 2018 ▸). Only in rare experimental designs, such as crystallographic fragment screening (Pearce *et al.*, 2017 ▸), does the absence of ligand density provide useful information.

### 1.3. Density myopia

The situation is different in the case of covalently bound parts of the molecule that are *known to be present*, likely as an ensemble of multiple conformations, but are not distinctly (or frequently not at all) visible in the resulting averaged electron density. Typical examples are protein chain termini, disordered side chains, loops between secondary structural elements, protein-linked glycosyl­ations or similar decorations, and even entire disordered protein domains. The model then is clearly missing something that must be present in the crystal structure (rare exceptions are cases where those parts of the molecule are cleaved away).

## 2. Suboptimal approaches to modelling missing parts

**Figure 1:**
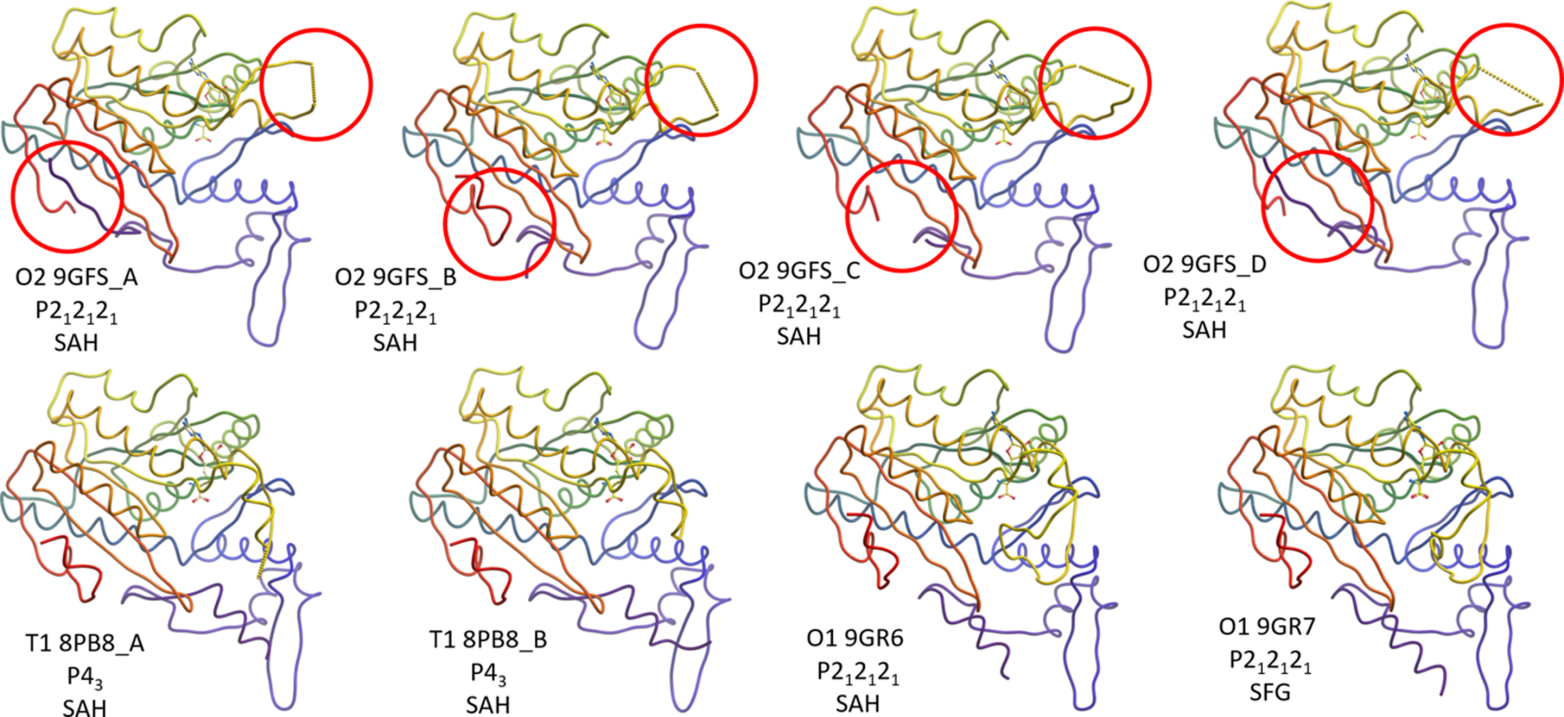
Eight different structure models of the fungal methyl­transferase PsiM, in three different crystal forms (*cf*. Table 1 ▸), each presented as a protein worm, coloured from the N-terminal (blue) to the C-terminal (red). The ball and stick models are the SAH and SFG coenzyme analogues. While we can see that the substrate recognition loop (SRL, top red circles in the top row) is incomplete in the first six models, the O1 form models present this loop as continuous and on par with other model parts, because any dynamic information is missing. From multiple crystal forms we can already infer that the N- and C-termini (lower red circles) can assume packing- and environment-dependent conformations – even a distinct α-helix of the N-terminal expression tag is partly visible in the bottom four models, albeit without any dynamic information.

**Figure 2:**
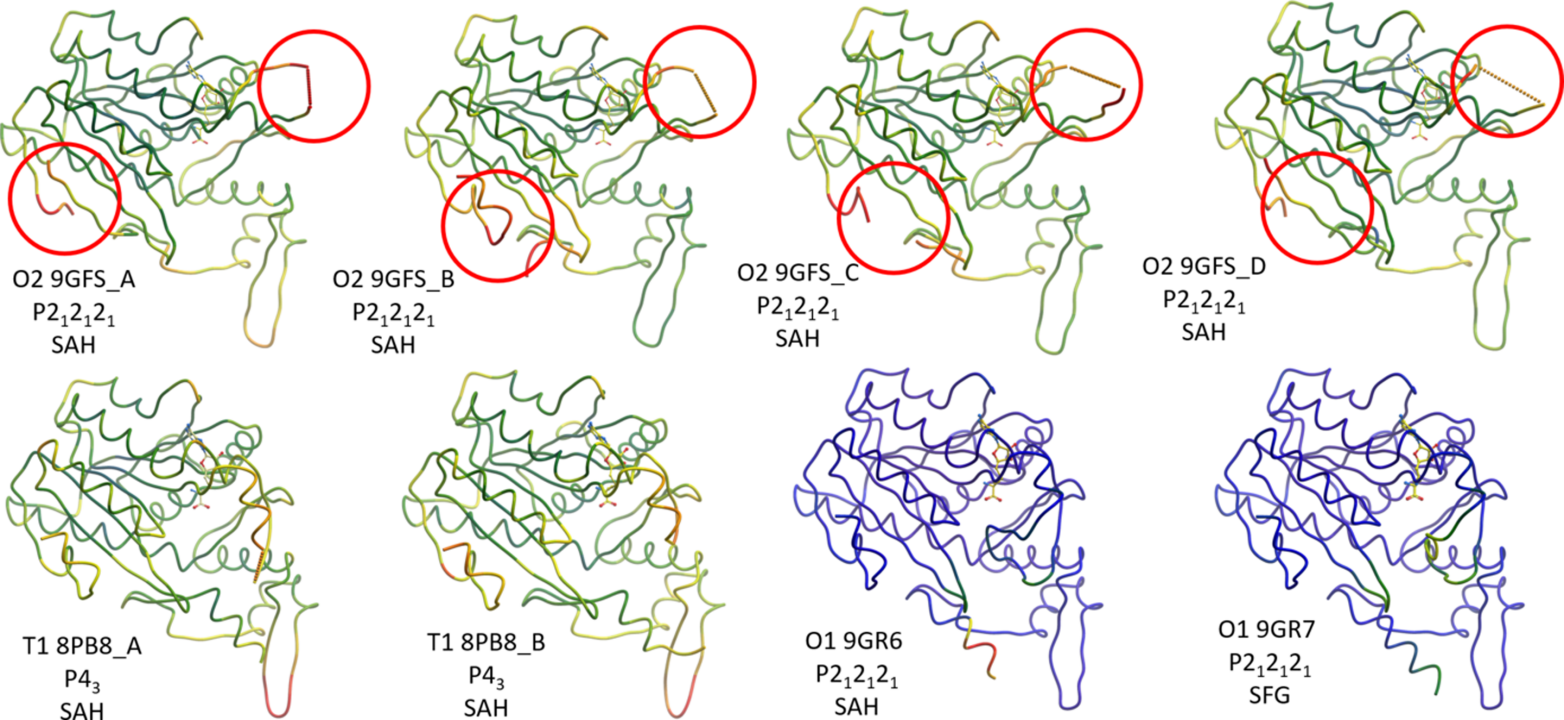
The same models as in Fig. 1 ▸ but coloured by the relative *B*-factor (blue – low, red – high; scale from 0 to 100 Å^2^). The dynamic nature of the still unmodelled termini and of the residues leading into the missing loop is partly reflected in this representation: The ‘hotter’ colour in the first six models for the termini and the residues leading into the loop immediately informs us that these areas have high *B*-factors and are probably disordered. Since the *B*-factor colours were chosen on an absolute scale, it is immediately apparent from this figure that the two O1 models (bottom right) were derived from data with much higher resolution compared with the first six models (*cf*. Table 1 ▸). Still, despite the hints from the elevated *B*-factor values also in the O1 models, we are missing information regarding the dynamics of the elusive specificity-determining loop of the methyl­transferase.

In the above case of covalently bound parts of the molecule, the model builder is faced with a dilemma: to model – or not to model – the invisible parts is the question. Regardless of the chosen path, almost all current practices are unsatisfactory.

(*a*) Simply do not model. This is often the case when large parts are missing and cannot be traced at all. It is an honest but unsatisfying approach, because the refinement programs then backfill these empty regions with disordered solvent, which is also not a correct description of the crystal structure.

(*b*) Use residue stubs (truncated side chains). Though stubs can be practical during early backbone building, the final model should not include amino acids that simply end at the β-carbon or at any other arbitrary end of the side chain. While admitting our level of ignorance about the positions of the remaining side-chain atoms, we know that stubs are not a correct model for a side chain.

(*c*) Divide the absent parts into one or two, maybe three, discrete conformations and set the atomic occupancies to zero. The refinement program excludes these atoms from the minimization, and thus will not refine the *B*-factors, which means the *B*-factors will remain at whatever arbitrary value they have been set to when building the model. In addition, generally no restraints will be applied and, as in the stub case, the solvent mask will extend over the zeroed atoms. The abuse of setting *near-zero* occupancies for ligands, thereby expelling the solvent mask and as a consequence generating artificial ligand-shaped solvent density, has been discussed (Wlodawer *et al.*, 2018 ▸). Finally, a display program will likely still show these atoms as ‘normal’ without a warning (Fig. 1 ▸). The zeroing method is probably the worst option and was revealing in a case of fabrication (Rupp, 2012 ▸).

(*d*) Intuit the absent parts in one or two conformations and simply refine. Looking at the structure factor formula (Blundell & Johnson, 1976 ▸; Rhodes, 2006 ▸; Rupp, 2009 ▸), one can infer what a refinement program’s response to this situation will be: with the incorrectly placed atoms tethered together by the bond restraints (preventing the refinement from sending the atoms into disordered solvent), the only remaining option for the refinement is to increase the *B*-factor to high values whereupon any unwanted scattering contribution of the high *B*-factor atoms becomes negligible. The *B*-factor, despite its possible interpretation as mean displacement (Willis & Pryor, 1975 ▸), is formally a simple parameter describing the probability of an atom being at its stated position, for whatever reason (Levin *et al.*, 2007 ▸). Historically, there seems to be a reluctance to let the *B*-factors run high (probably because the fixed Protein Data Bank (PDB) legacy format looks strange with *B*-factors at or above 100 Å^2^). However, if the *B*-factors are over-restrained, they may remain unjustifiably low and thus also lead to higher *R* values than for the more realistically relaxed *B*-factor restraints (Tronrud, 1996 ▸). While running up the *B*-factors is probably the most defendable option, a display program will still show the model only in one, or maybe two, built conformations, and an unsuspecting user may not recognize the associated high *B*-factors (Fig. 2 ▸).

## 3. Exploring the void

At this point, one may ask – why does it matter whether and how we model ‘absent’ parts of a crystal structure? Either way, by omitting them or by accepting high *B*-factors, we simply acknowledge that we have very limited or no direct evidence for the presence of these parts of the structure. But multiple, flexible or missing sections can have functional relevance, particularly when large parts, such as flexible loops or domains, exercise a function precisely *because* they possess a large degree of conformational flexibility. Ignoring such ‘invisible’ regions completely, without providing any indication where they might go, is unsatisfactory and leaves the model incomplete.

The question of where missing parts might go becomes even more challenging when considering that the flexible regions are not in a native solution environment, but their conformational space is restricted by crystal packing. Different crystal packing forces the missing parts to explore different conformational spaces. Similarly, in crystal structures containing multiple non-crystallographic symmetry (NCS) related copies in the asymmetric unit, the conformational space available for each protomer can be significantly different.

## 4. Modelling the unknown

The challenge of correctly representing flexible parts of a molecule has been recognized as partly responsible for the large gap between the data quality (data-merging residuals) and the generally much higher model refinement residuals (Holton *et al.*, 2014 ▸). Ensemble refinement (ER) (Levin *et al.*, 2007 ▸; Burnley *et al.*, 2012 ▸) allows for more realistic modelling of flexibility in crystal structures through simultaneous time-averaged refinement of a set of multiple models combining molecular dynamics (MD) with an X-ray target: the computational modelling based on MD potentials (Kuriyan *et al.*, 1991 ▸; Moriarty *et al.*, 2020 ▸; Wych *et al.*, 2023 ▸) is kept in the confines of reality by the X-ray terms. Local molecular vibrations are sampled by MD simulation, and global disorder is modelled with a translation–libration–screw model (Burnley *et al.*, 2012 ▸). Instead of generating ensembles of independent models, multi-conformer refinement (MCR) takes a slightly different approach (Wankowicz *et al.*, 2024 ▸) by representing the distribution of states contributing to the average density map with altloc identifiers in the ATOM records where needed. It is important to understand the result of ER exactly as what is intended: the entire ensemble of models provides a description of reality. Extracting any single individual model from the set is generally not meaningful.

Both ER and MCR have been successfully applied to reveal functional significance of mostly local molecular plasticity. In the following, we show that ER of complete models does also allow for visualization and exploration of the available conformation space of large, entirely ‘invisible’ regions of a crystal structure. Combined with different crystal forms, insights into functional aspects of molecular plasticity can be inferred. Though the parametrization of ER to create model ensembles for PDB deposition can be challenging (Burnley & Gros, 2013 ▸), models suitable for the exploration of protein dynamics can be easily obtained using *Phenix* (Adams *et al.*, 2010 ▸) default settings.

## 5. A magic example

**Figure 3:**
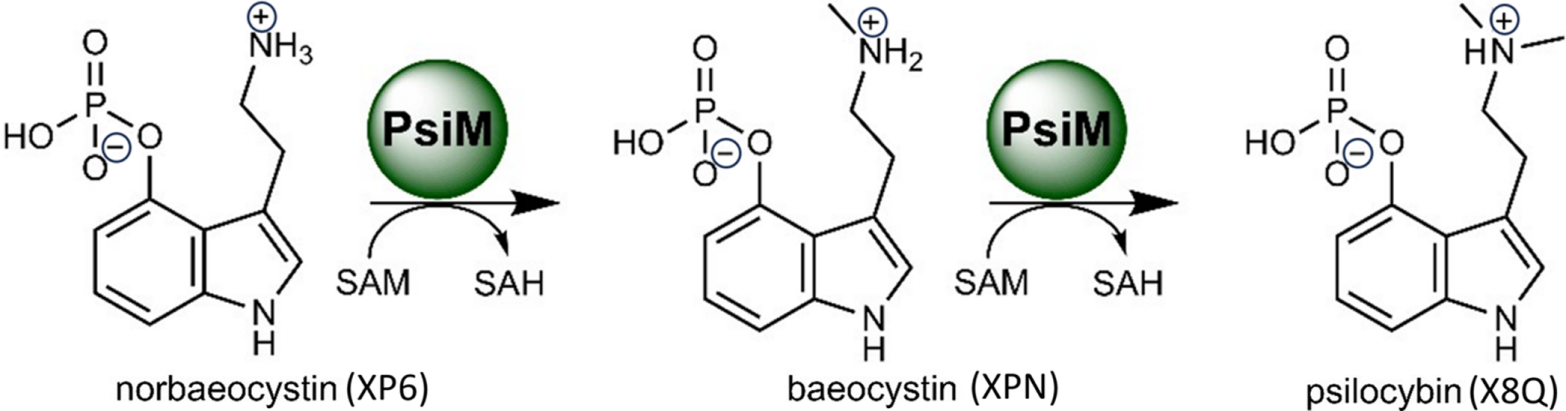
Methyl­ation reaction sequence. The fungal methyl­transferase PsiM uses SAM as a methyl source to process XP6 into XPN, and in a second step XPN into the hallucinogen X8Q. Figure adapted from the work by Hudspeth *et al.* (2024*a* ▸), licenced under http://creativecommons.org/licenses/by/4.0/.

The two ultimate methyl­ation steps in the biosynthesis of the hallucinogen psilocybin (X8Q) by various magic mushrooms (Fricke *et al.*, 2018 ▸) from norbaeocystin (XP6) via baeocystin (XPN) to X8Q are successively carried out (Fig. 3 ▸) by the same fungal methyl­transferase, PsiM, with the coenzyme *S*-adenosylme­thio­nine (SAM) acting as the methyl donor (Fricke *et al.*, 2017 ▸).

The details of the actual methyl-transfer mechanism from the non-covalently bound SAM to XP6 and XPN in PsiM from *Psilocybe cubensis* have been elucidated from numerous ternary coenzyme–substrate–enzyme structures (Hudspeth *et al.*, 2024*a* ▸; Hudspeth *et al.*, 2024*b* ▸), while the dynamics of reloading PsiM with SAM are still speculative. Characteristic is the absence of a SAM-free apo structure indicating that the SAM loading is a highly dynamic process during which the molecule presumably partly unfolds and thus, due to the resulting conformational disorder, cannot be crystallized. However, a set of seven structure models of the SAH-bound – but substrate-free – structures in different crystal forms are available, where the dynamic behaviour of a unique, 32-residue substrate recognition loop (SRL, residues 189–221) provides the first clues to the substrate-loading process.

**Figure 4:**
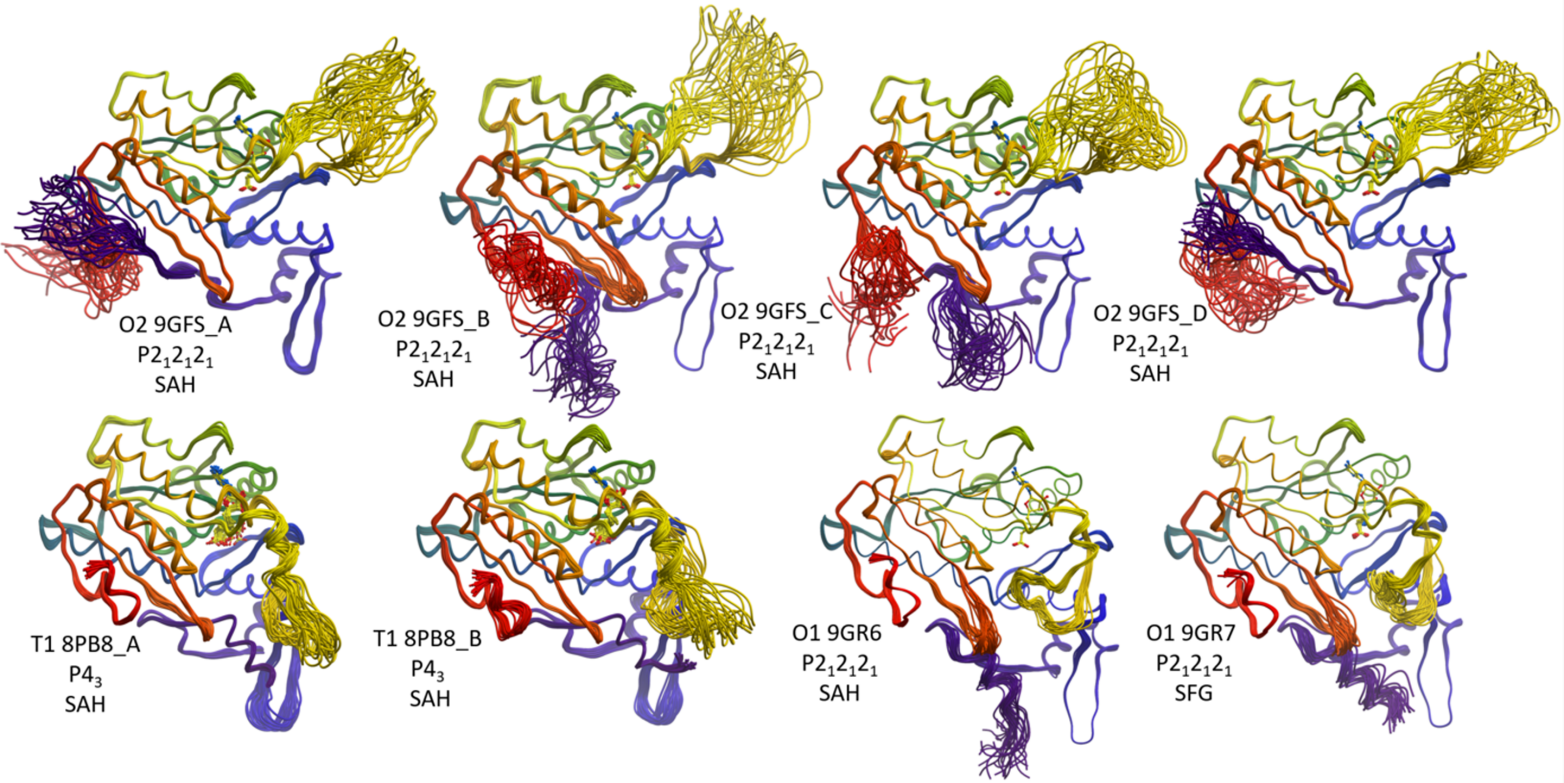
Visualization of missing parts through ER. The same models as in the previous figures, this time as a set of 25 ER models, coloured from N- to C-termini. In the top row, the four NCS-related copies show that the SRL (yellow loop) can extend in a highly disordered manner into a solvent void, in a similar fashion for each protomer. In contrast, the conformation of the termini is different in each of the four copies due to the different packing contacts and available void space. In the T1 apo structure models (bottom left), the termini assume a distinct, packing-induced secondary helical structure, while the SRL now folds back covering the entrance to the empty substrate-binding site. Even for the two atomic-resolution structure models (bottom right) where the absolute *B*-factor scale coloured models in Fig. 2 ▸ suggested a rigid N-terminal helix, the dynamic nature of this extension becomes evident in the ensemble models.

Each panel of Fig. 4 ▸ shows the 25 PsiM models resulting from ER of the respective completed PsiM starting model. All missing residues were built in an arbitrary idealized conformation into available void (solvent) space of the published PDB models (Table 1 ▸), and the resulting models underwent ER in *Phenix* (*cf*. *Methods*[Sec sec7]). The ensemble models show the previously missing termini and SRL exploring the available conformation space. In the four NCS-related copies, the SRL is highly disordered and explores a solvent void, while the conformation of the termini differs between the copies due to different packing contacts and different available free space. In the T1 and O1 apo structure models, the termini assume a distinct, packing-induced secondary and mostly helical structure at the N-terminal. The SRL now folds back covering the entrance to the empty substrate-binding site.

From the ER models we can conclude that the SRL, despite appearing relatively well ordered and rigid in the O1 high-resolution structures, is genuinely flexible and can assume a wide array of conformations in the absence of substrate. The overall picture indicates that a basic open–close ‘flap’ mechanism is probably an oversimplification.

**Figure 5:**
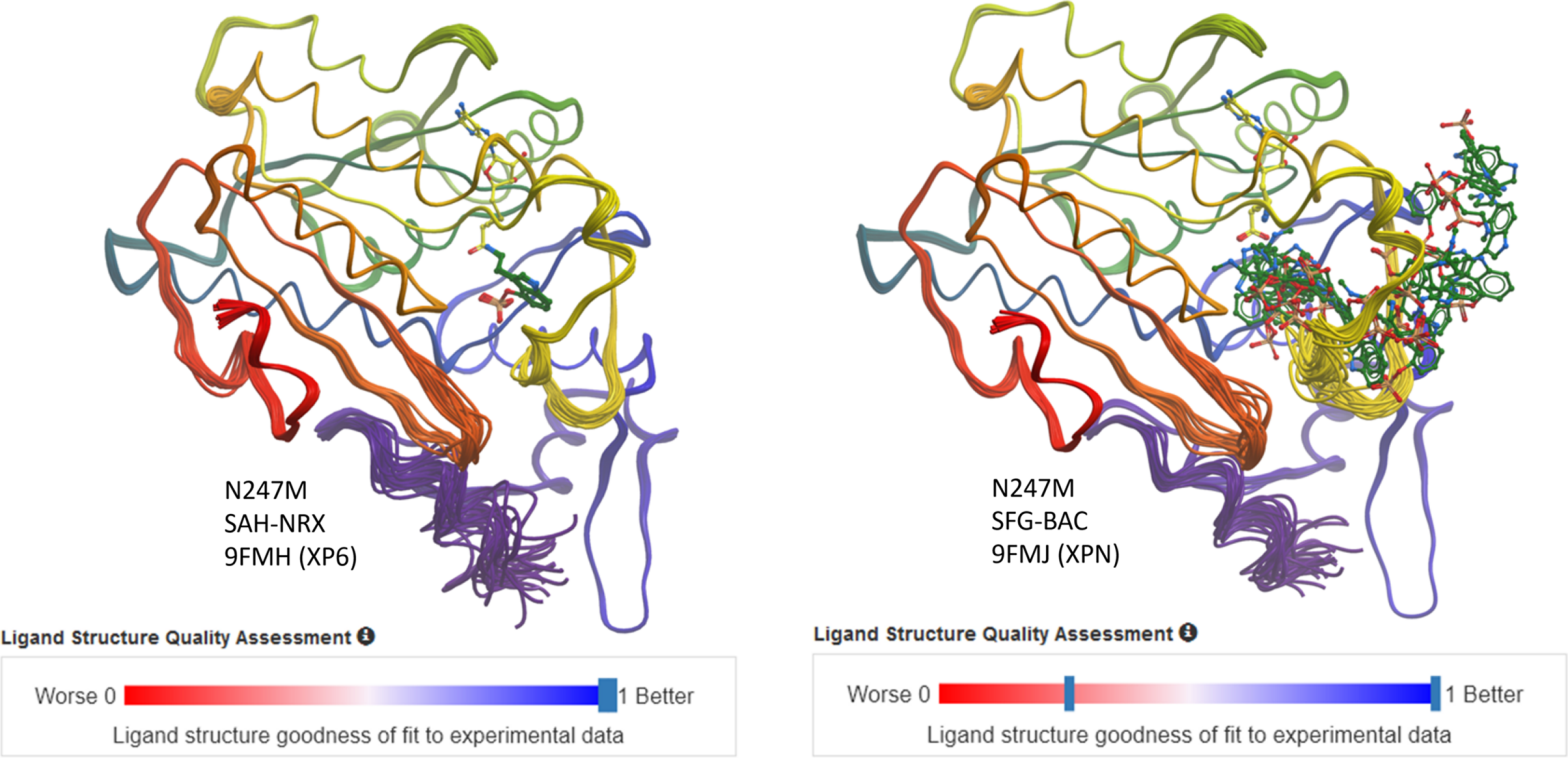
Ligand validation via ER. In the ensemble model of PsiM mutant N247M, the bound XP6 (left panel) shows very little pose variation in agreement with good real space correlation and RSR. In contrast, the tentatively modelled, weakly bound XPN (right panel) can depart the binding site through the mobile substrate-recognition loop. The strong and weak ligand binding is also indicated in the respective PDB ligand sliders. Likewise, note the higher ligand flexibility (also for XP6) compared with the rigidly bound coenzymes SAH and SFG.

ER can also be used to visualize ligand dynamics (Caldararu *et al.*, 2021 ▸). In a previous study (Hudspeth *et al.*, 2024*b* ▸) focused on elucidating the effects of second-shell coordination on substrate binding, it was found that the N247M PsiM mutant binds the substrate XP6 for the first methyl­ation tightly, while in the same mutant, the binding of the second methyl­ation substrate XPN is poor and its modelling was uncertain. The tight binding of XP6 is clearly reflected in the ER: the XP6 molecule remains in a practically identical position in all models (Fig. 5 ▸). ER of the tentatively modelled XPN ligand delivers an entirely different result: XPN can depart the binding site through the widened opening in the SRL. This outcome indicates that a long-range movement of the SRL ‘flap’, as suggested by the variability of this loop in unbound apo models (Fig. 4 ▸), might not be a strict requirement for substrate loading and product release.

**Figure 6:**
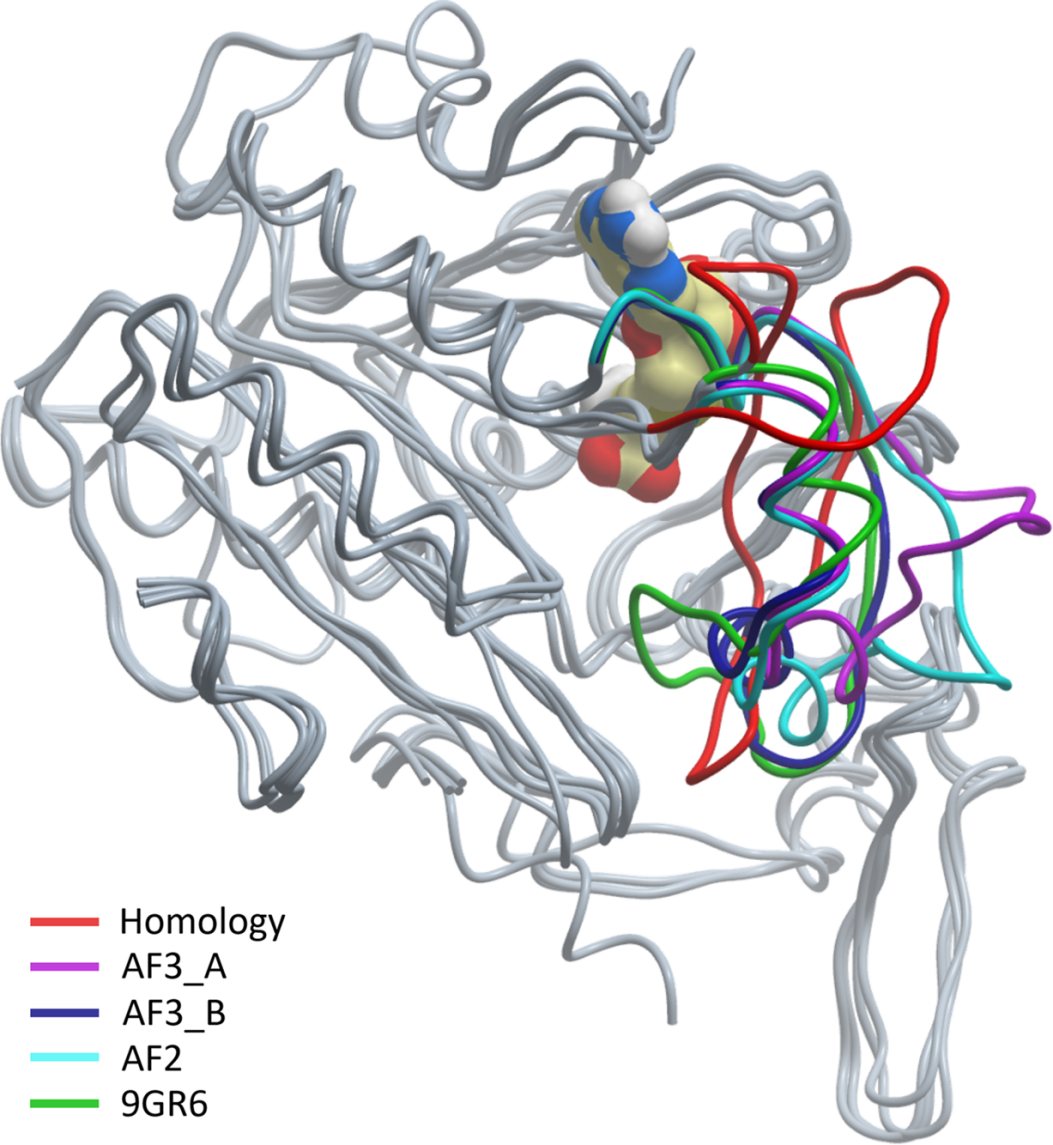
Superposition of computational models with the O1 form crystal structure model of PsiM. In the absence of an anchoring template beyond the Rossman fold enzyme core, the pre-*AF* homology model (red) delivers a random conformation for the SRL, while *AF*2 (cyan) provided with low confidence a similarly unstructured SRL conformation. *AF*3 delivered two slightly different models, one with an open-loop conformation (purple) while the alternative *AF*3 model (blue) partly follows the experimental O1 model (green). As is the case for X-ray models, the ensemble of various computational models emphasizes that the uncertainties of the models in the SRL are larger than each single prediction model suggests.

We also wish to caution against the perception that *AlphaFold* (*AF*) machine-learning models (Jumper *et al.*, 2021 ▸) will make experimental structure determination almost superfluous (Terwilliger *et al.*, 2024 ▸). Irrespective of their undisputed value for providing starting models for experimental techniques (Terwilliger *et al.*, 2022 ▸), the insights into the problem of ‘invisible’ regions that a single *AF* model can provide are as limited as for a single X-ray model. The PsiM case can serve as an illustrative example here as well. Fig. 6 ▸ compares a pre-*AF* homology model (Fricke *et al.*, 2019 ▸), an *AF*2 model *before* the crystal structure was deposited and *AF*3 models *after* the experimental structures were deposited in March 2024, with the actual crystal structure of PsiM complexed with SAH in its high-resolution O1 form (Hudspeth *et al.*, 2024*a* ▸).

A qualitative inspection of Fig. 6 ▸ already shows that the accuracy of the prediction models has dramatically increased from the pre-*AF*, template-dependent homology model to the *AF*2 model (which was unaware of the X-ray models) and then to the *AF*3 models. As in the X-ray case, a single computational model can still deliver a plausible conformation, and only the low-confidence indicators of the SRL region warn the user that high conformational flexibility exists. Again, it is the ensemble of prediction models in Fig. 6 ▸ that conveys that the SRL is likely to be more flexible than any single model can suggest. Some bias of the *AF*3 models towards already deposited templates exists, likely leading to propagation of conformations induced by crystal packing as is the case of the O1 model (the option to exclude PDB templates does exist in *AF*3).

## 6. Conclusions

Dynamic processes such as substrate loading in the PsiM example are difficult to explore by crystallography. The commonly used static models and their depictions rarely do justice to the dynamic nature of protein molecules, and the same limitations apply to purely computational models: one *AF* model will not provide a complete picture of the dynamics of the underlying molecule. We suggest using ER as a powerful exploratory tool suitable for the effective visualization of crystal structure models. Particularly when using ER on multiple crystal forms, the visual persuasiveness of the structural plasticity and its context sensitivity are often striking and deliver a more ‘holistic’ representation of the molecule dynamics. In addition, applying ER to ligand structure models provides a vivid visualization of conformational rigidity or flexibility of a bound ligand.

## 7. Methods

Model coordinates for PsiM (Table 1 ▸) were extracted from the Protein Data Bank (wwPDBconsortium, 2019 ▸). The missing parts of the models were completed according to the protein construct sequence using *Coot* (Emsley & Cowtan, 2004 ▸; Casañal *et al.*, 2020 ▸) in a single idealized conformation extending into the empty solvent region. Multi-conformer refinement of 25 models of each completed entry (Hudspeth *et al.*, 2024*a* ▸; Hudspeth *et al.*, 2024*b* ▸) was carried out with the *phenix.ensemble_refinement* module of *Phenix* (Adams *et al.*, 2010 ▸; Moriarty *et al.*, 2020 ▸) in the default settings. The figures were generated with Molsoft *ICM BrowserPro* (https://www.molsoft.com/icm_browser_pro.html) and composed in Microsoft *PowerPoint*.

## Figures and Tables

**Table 1:** Previously published PsiM–SAH/SFG complex models used for ER

PDB code	Crystal form	Space group	Resolution (Å)	N in ASU	Coenzyme	Ligand
8pb8	T1	*P*4_3_	2.53	2	SAH	–
9gfs	O2	*P*2_1_2_1_2_1_	1.98	4	SAH	–
9gr6	O1	*P*2_1_2_1_2_1_	0.93	1	SAH	–
9gr7	O1	*P*2_1_2_1_2_1_	1.20	1	SFG	–
9fmh	O1	*P*2_1_2_1_2_1_	0.90	1	SAH	XP6
9fmj	O1	*P*2_1_2_1_2_1_	0.95	1	SFG	XPN

ASU: asymmetric unit; SAH: *S*-adenosylhomocysteine; SFG: sinefungin, a non-processible SAM analogue; XP6: PDB-assigned heterogen code for norbaeocystin; XPN: PDB-assigned heterogen code for baeocystin.

## Data Availability

The discussed PsiM models have been previously deposited with the PDB (8pb8, 9gfs, 9gr6, 9gr7, 9fmh, 9fmj).

## References

[bb1] Adams, P. D., Afonine, P. V., Bunkóczi, G., Chen, V. B., Davis, I. W., Echols, N., Headd, J. J., Hung, L.-W., Kapral, G. J., Grosse-Kunstleve, R. W., McCoy, A. J., Moriarty, N. W., Oeffner, R., Read, R. J., Richardson, D. C., Richardson, J. S., Terwilliger, T. C. & Zwart, P. H. (2010). *Acta Cryst.* D**66**, 213–221. 10.1107/S0907444909052925 PMC2815670 20124702

[bb2] Bacon, F. (1620). *Novum organum scientarium; partis secundae summa, digesta in aphorismos*, Aphorismus XLIX.

[bb3] Blundell, T. L. & Johnson, L. N. (1976). *Protein crystallography*. Academic Press.

[bb4] Burnley, B. T., Afonine, P. V., Adams, P. D. & Gros, P. (2012). *Elife*, **1**, e00311. 10.7554/eLife.00311 PMC3524795 23251785

[bb5] Burnley, B. T. & Gros, P. (2013). *Comput. Crystallogr. Newsl.* **4**, 51–58.

[bb6] Caldararu, O., Ekberg, V., Logan, D. T., Oksanen, E. & Ryde, U. (2021). *Acta Cryst.* D**77**, 1099–1115. 10.1107/S2059798321006513 PMC8329865 34342282

[bb7] Casañal, A., Lohkamp, B. & Emsley, P. (2020). *Protein Sci.* **29**, 1069–1078. 10.1002/pro.3791 PMC7096722 31730249

[bb8] Emsley, P. & Cowtan, K. (2004). *Acta Cryst.* D**60**, 2126–2132. 10.1107/S0907444904019158 15572765

[bb9] Fricke, J., Blei, F. & Hoffmeister, D. (2017). *Angew. Chem. Int. Ed.* **56**, 12352–12355. 10.1002/anie.201705489 28763571

[bb10] Fricke, J., Lenz, C., Wick, J., Blei, F. & Hoffmeister, D. (2018). *Chem. A Eur. J.* **25**, 897–903. 10.1002/chem.201802758 30011099

[bb11] Fricke, J., Sherwood, A., Kargbo, R., Orry, A., Blei, F., Naschberger, A., Rupp, B. & Hoffmeister, D. (2019). *ChemBioChem*, **20**, 2824–2829. 10.1002/cbic.201900358 31150155

[bb12] Holton, J. M., Classen, S., Frankel, K. A. & Tainer, J. A. (2014). *FEBS J.* **281**, 4046–4060. 10.1111/febs.12922 PMC4282448 25040949

[bb13] Hudspeth, J., Rogge, K., Dörner, S., Müll, M., Hoffmeister, D., Rupp, B. & Werten, S. (2024*a*). *Nat. Commun.* **15**, 2709. 10.1038/s41467-024-46997-z PMC10978996 38548735

[bb14] Hudspeth, J., Rogge, K., Wagner, T., Mull, M., Hoffmeister, D., Rupp, B. & Werten, S. (2024*b*). *Chembiochem*, **25**, e202400497. 10.1002/cbic.202400497 39413044

[bb15] Jumper, J., Evans, R., Pritzel, A., Green, T., Figurnov, M., Ronneberger, O., Tunyasuvunakool, K., Bates, R., Žídek, A., Potapenko, A., Bridgland, A., Meyer, C., Kohl, S. A. A., Ballard, A. J., Cowie, A., Romera-Paredes, B., Nikolov, S., Jain, R., Adler, J., Back, T., Petersen, S., Reiman, D., Clancy, E., Zielinski, M., Steinegger, M., Pacholska, M., Berghammer, T., Bodenstein, S., Silver, D., Vinyals, O., Senior, A. W., Kavukcuoglu, K., Kohli, P. & Hassabis, D. (2021). *Nature*, **596**, 583–589. 10.1038/s41586-021-03819-2 PMC8371605 34265844

[bb16] Kleywegt, G. J. & Jones, T. A. (1995). *Structure*, **3**, 535–540. 10.1016/s0969-2126(01)00187-3 8590014

[bb17] Kuriyan, J., Ösapay, K., Burley, S. K., Brünger, A. T., Hendrickson, W. A. & Karplus, M. (1991). *Proteins*, **10**, 340–358. 10.1002/prot.340100407 1946343

[bb18] Levin, E. J., Kondrashov, D. A., Wesenberg, G. E. & Phillips, G. N. (2007). *Structure*, **15**, 1040–1052. 10.1016/j.str.2007.06.019 PMC2039884 17850744

[bb19] Moriarty, N. W., Janowski, P. A., Swails, J. M., Nguyen, H., Richardson, J. S., Case, D. A. & Adams, P. D. (2020). *Acta Cryst.* D**76**, 51–62. 10.1107/S2059798319015134 PMC6939439 31909743

[bb20] Pearce, N. M., Krojer, T., Bradley, A. R., Collins, P., Nowak, R. P., Talon, R., Marsden, B. D., Kelm, S., Shi, J., Deane, C. M. & von Delft, F. (2017). *Nat. Commun.* **8**, 15123. 10.1038/ncomms15123 PMC5413968 28436492

[bb21] Pozharski, E., Weichenberger, C. X. & Rupp, B. (2013). *Acta Cryst.* D**69**, 150–167. 10.1107/S0907444912044423 23385452

[bb22] Rhodes, G. (2006). *Crystallography made crystal clear*, 3rd ed. Academic Press.

[bb23] Rupp, B. (2009). *Biomolecular crystallography: principles, practice, and application to structural biology*, 1st ed. Garland Science.

[bb24] Rupp, B. (2012). *Acta Cryst.* F**68**, 366–376. 10.1107/S1744309112008421 PMC3325800 22505400

[bb25] Terwilliger, T. C., Liebschner, D., Croll, T. I., Williams, C. J., McCoy, A. J., Poon, B. K., Afonine, P. V., Oeffner, R. D., Richardson, J. S., Read, R. J. & Adams, P. D. (2024). *Nat. Methods*, **21**, 110–116. 10.1038/s41592-023-02087-4 PMC10776388 38036854

[bb26] Terwilliger, T. C., Poon, B. K., Afonine, P. V., Schlicksup, C. J., Croll, T. I., Millán, C., Richardson, J. S., Read, R. J. & Adams, P. D. (2022). *Nat. Methods*, **19**, 1376–1382. 10.1038/s41592-022-01645-6 PMC9636017 36266465

[bb27] Tronrud, D. E. (1996). *J. Appl. Cryst.* **29**, 100–104.

[bb28] Wankowicz, S. A., Ravikumar, A., Sharma, S., Riley, B., Raju, A., Hogan, D. W., Flowers, J., van den Bedem, H., Keedy, D. A. & Fraser, J. S. (2024). *Elife*, **12**, RP90606. 10.7554/eLife.90606 PMC11192534 38904665

[bb29] Willis, B. T. M. & Pryor, A. W. (1975). *Thermal vibrations in crystallography*. Cambridge University Press.

[bb30] Wlodawer, A., Dauter, Z., Porebski, P. J., Minor, W., Stanfield, R., Jaskolski, M., Pozharski, E., Weichenberger, C. X. & Rupp, B. (2018). *FEBS J.* **285**, 444–466. 10.1111/febs.14320 PMC5799025 29113027

[bb31] wwPDBconsortium (2019). *Nucleic Acids Res.* **47**, D520–D528. 10.1093/nar/gky949 PMC6324056 30357364

[bb32] Wych, D. C., Aoto, P. C., Vu, L., Wolff, A. M., Mobley, D. L., Fraser, J. S., Taylor, S. S. & Wall, M. E. (2023). *Acta Cryst.* D**79**, 50–65. 10.1107/S2059798322011871 PMC9815100 36601807

